# Developmental Perspectives on Nutrition and Obesity From Gestation to Adolescence

**Published:** 2009-06-15

**Authors:** Terry T. Huang, Layla Esposito, Jennifer O. Fisher, Julie A. Mennella, Deanna M. Hoelscher

**Affiliations:** Eunice Kennedy Shriver National Institute of Child Health and Human Development; Eunice Kennedy Shriver National Institute of Child Health and Human Development, Bethesda, Maryland; Temple University, Philadelphia, Pennsylvania; Monell Chemical Senses Center, Philadelphia, Pennsylvania; University of Texas School of Public Health, Houston, Texas

## Abstract

Obesity results from a complex combination of factors that act at many stages throughout a person's life. Therefore, examining childhood nutrition and obesity from a developmental perspective is warranted. A developmental perspective recognizes the cumulative effects of factors that contribute to eating behavior and obesity, including biological and socioenvironmental factors that are relevant at different stages of development. A developmental perspective considers family, school, and community context. During gestation, risk factors for obesity include maternal diet, overweight, and smoking. In early childhood, feeding practices, taste acquisition, and eating in the absence of hunger must be considered. As children become more independent during middle childhood and adolescence, school nutrition, food marketing, and social networks become focal points for obesity prevention or intervention. Combining a multilevel approach with a developmental perspective can inform more effective and sustainable strategies for obesity prevention.

## Introduction

Obesity results from a combination of factors that occur at different stages during a person's lifetime. Therefore, childhood nutrition and obesity should be examined from a developmental perspective. First, prenatal and early life experiences influence the trajectory of weight into adulthood (1). Second, during certain critical periods, vulnerabilities are intensified to specific maternal and environmental exposures that can lead to obesity (2). Finally, the cumulative effects of multiple factors contribute to eating behavior and obesity (3).

Growing evidence suggests that prenatal and maternal interactions and influences must be considered along with biological and environmental variables throughout infancy, childhood, and adolescence that may lead to — or prevent — obesity. Examining nutrition and obesity from a developmental perspective combines social context and biological influences with individual behavior (4,5). Social context can range from family, to school, to the broader community. We describe where these contexts interact with biological processes to affect food behavior and obesity. Although a person is at risk for obesity throughout his life, we focus on specific developmental susceptibilities for obesity from gestation through adolescence (Table).

## Gestational Period

Risk for obesity and metabolic disorders begins during gestation (1). Obesity is linked to in utero exposure to glucocorticoids, protein restrictions, and maternal diet and obesity. Exposing fetal rats to high levels of glucocorticoids reduces birth weight and results in adults with high blood levels of insulin and glucose. Male offspring of female rats with a history of fetal exposure to glucocorticoids also exhibit low birth weight and glucose intolerance — a multigenerational effect (6).

Feeding low-protein diets to pregnant rats produces a broad spectrum of disorders in their offspring (7): hypertension and vascular defects (8,9), altered fetal pancreatic development and structure (10), altered glucose tolerance (11), altered liver structure and function (12), altered gene expression (13), and possibly type 2 diabetes mellitus (10). In humans, low protein intake by women in late pregnancy has been associated with low birth weight, a marker of risk for obesity and other metabolic disorders later in life (14).

A maternal diet high in fat also causes long-term harm to the offspring. Female rat pups born to and suckled by fat-fed mothers have high blood pressure, even after being placed on a balanced diet after weaning. The offspring are hypertensive, show vascular changes, and have high blood insulin levels. Such changes in early life are likely to lead to metabolic syndrome in adult animals (15).

Many studies have indicated a link between smoking during pregnancy and the offspring's subsequent obesity, but the underlying mechanism has not been established. Children born to women who smoke during pregnancy typically weigh less at birth, and they often have a catch-up period during their first year, although studies have not consistently found a link between catch-up growth and greater childhood body mass index (BMI). Other hypotheses postulate mechanisms such as poor placental blood supply because of nicotine-induced vasoconstriction, poor maternal nutrition, and fetal exposure to carbon monoxide. Whatever the mechanism, the relationship between smoking during pregnancy and children's overweight is well documented (16,17)

In 1 study, for example, babies born to mothers who smoked during pregnancy weighed less than did babies born to nonsmokers (18). However, as they reached adolescence (age 11 years for girls, 16 for boys), children exposed to tobacco in utero had a significantly greater risk of being in the highest 10% of BMI for their age group. This tendency continued to strengthen with age (participants were followed through age 33) and could not be explained by other factors in their childhood, adolescence, or adulthood (18).

A recent 27-year study of children whose mothers smoked during pregnancy found larger annual changes in cholesterol levels; high-density lipoprotein cholesterol levels decreased and low-density lipoprotein cholesterol levels increased more than in children not exposed to tobacco in utero. This was the first study to suggest that smoking during pregnancy is linked to adverse changes in the lipoprotein levels of children (19). In an analysis of questionnaire data from 8,765 children aged 5 to 7 years, smoking after pregnancy was not associated with childhood obesity but intrauterine exposure was (20). Another study found that smoking during the 12 months before birth of a child was associated with adolescent overweight (21).

Studies have found a significant association between maternal prepregnancy overweight or obesity and overweight in children. This association indicates that overweight mothers are more likely to have overweight children, and these odds increase with the age of the child. For children aged 24 to 47 months, only maternal prepregnancy obesity had a significant effect; for children aged 48 to 71 months, either maternal prepregnancy overweight or obesity increased risk; and in children aged 72 to 95 months, maternal overweight or obesity imparted an even higher risk (21,22). Breast-feeding reduced the likelihood of early adolescent overweight in children whose mothers' prepregnancy BMI was 25 or higher, although the effect of breast-feeding was not significant in children of healthy-weight mothers (21,22). Thus, both prenatal and maternal variables can increase the risk of obesity in even the youngest children, long before social factors have an influence. Obesity prevention efforts at this stage of development have typically focused on encouraging healthy prenatal nutrition and breast-feeding; however, interventions to reduce maternal obesity during pregnancy have been limited.

## Infancy and Early Childhood

### Taste acquisition and preference

The biological substrate that underlies the taste and rewarding properties of foods is relevant because the best predictor of food preference is whether a child likes the taste (23). Whether a food tastes good or bad and the pleasure of eating is a complex process mediated by chemical senses in the periphery and multiple brain substrates, which are remarkably well conserved phylogenetically (24).

The degree to which taste and smell are agreeable is determined by innate factors (25), learning and experience, and the interactions among these. From an evolutionary perspective, these senses, which are well developed at birth, function as gatekeepers throughout life (26). The small number of taste qualities may have evolved because of the functional importance of the primary stimuli (eg, sugars, sodium chloride, bitter toxins) in nutrient selection, especially in children. The heightened preference for sweet taste, which is evident within hours after birth and persists until adolescence (27,28) most likely evolved because sweet-tasting foods are high in energy. Children's heightened preference for salty tastes (29) attracts them to necessary minerals, and rejecting bitter-tasting substances protects them from poisons because most poisonous compounds taste bitter (30). However, although bitter tastes are innately disliked, with repeated exposure, infants can come to like certain foods that are bitter, particularly some vegetables (31-34).

The 2002 Feeding Infants and Toddlers Study, designed to update knowledge on the feeding patterns of the youngest Americans, found that even before their second birthday, many American toddlers develop the unhealthy eating habits of adults (35). Although toddlers were more likely to eat fruits than vegetables, 1 in 4 did not eat any vegetables on a given day. Instead, like older children, they were more likely to eat fatty foods such as french fries, salty snacks, and sweet beverages and less likely to eat bitter-tasting vegetables (36,37). None of the top 5 vegetables eaten by toddlers was a dark green vegetable.

Our knowledge is growing of how, beginning very early in life, early sensory experience can shape and modify flavor and food preferences. For example, fetuses exposed to flavors, usually detected by the sense of smell, in amniotic fluid and infants exposed to flavors in breast milk (both of which reflect flavors of the mother's diet) (38) learn to like those flavors as they make the transition to eating adult foods (39). The foods that women eat when they are pregnant and nursing are precisely the ones that their infants should prefer because the mothers' eating them teaches the child that these foods are available, safe, and nutritious. At this time, however, how the protective factor of breast-feeding interacts with transmitting flavor preference for energy-dense foods in overweight mothers is unclear.

These sensory and biological considerations shed light on why lifestyle changes are difficult for young children to make. We cannot easily change the basic ingrained biology of liking sweets and avoiding bitterness — preferring candy to spinach. What we can do is modulate children's flavor preferences by providing early exposure, starting in utero and early infancy, to a variety of healthy flavors. The first emotional attachment to flavors should be exploited to try to reduce the prevalence of obesity in future generations. For this reason, preventive interventions may be most effective during pregnancy and postpartum, when women are highly motivated to change for the benefit of their children. Pregnant and lactating women should widen their food choices to include as many flavorful and healthy foods as possible. These experiences, combined with repeated exposure to nutritious foods and flavor variety (31-34), should make children more likely to choose a healthy diet.

### Eating in the absence of hunger

Infants (40,41) and young children (42,43) can adjust their food intake in response to changes in the caloric content of their diet, and biological sensations involving appetite probably underlie this ability. This ability has been documented at meals (42,43) and during the course of a day (44). The complex interaction of nature and nurture in the regulation of appetite (45) is exemplified by a behavior known as eating in the absence of hunger (EAH), a behavioral marker of impaired satiety (46-49). Children ranging in age from 3 (50) to 19 years (51) have been observed in laboratory settings to eat large amounts of palatable food in the absence of hunger, after a meal. The amount of energy consumed in the absence of hunger is variable and related to child weight. EAH is seen more often in children who are overweight (39,47,49,52) and in children with higher 1-year weight gains (53). This behavior is analogous to external (53) or disinhibited (54) eating behavior in adults.

Genes influence many aspects of eating behavior, including taste sensitivity (55), food preference (56), intake of specific foods (57), meal patterns (58), energy density (59), macronutrient intake (60,61), and meal (61-63) and daily energy intake (61). Behaviors such as EAH may also be heritable (64), although evidence is limited. Genetic influences on EAH (49,65) and other eating behaviors such as emotional eating (66) are supported by findings that the behavior is more common when 1 or both parents are overweight, even after certain environmental factors (eg, parental eating habits) have been controlled for. Evidence for genetic influence on intake regulation is also reflected in the relative stability of behaviors like EAH within people during 2- to 6-year periods in childhood (47,67).

The biological underpinnings of EAH and other appetite-related behaviors are not well understood. EAH in children is associated with higher fasting insulin and leptin levels (64), 2 hormones that regulate appetite and body weight (68). Satiety responsiveness, a separate dimension of child intake regulation, has recently been linked to variations in the *FTO* gene (69), which confers obesity risk and is highly expressed in the hypothalamus, a center of appetite regulation in the brain.

Similar to other aspects of appetite regulation (70,71), EAH appears to become more problematic throughout childhood (45,72). Though the causes are not known, socioenvironmental influences contribute to developmental shifts in intake regulation by overriding biologically based cues of hunger and satiety. Factors that modify intake regulation include the types and amounts of food to which children are exposed, social modeling of eating behaviors, and child feeding styles and practices (73). For instance, experimental research has demonstrated social modeling influences on both the types (74) and amounts of food eaten by young children (75-77). Studies of EAH among girls have shown positive associations with mothers' but not fathers' disinhibited eating (52,65).

EAH has also been associated with restrictive feeding practices, although not consistently. Restricting children's access to a preferred food has been associated with higher levels of EAH in girls aged 3 to 5 years (49,78) and in non-Hispanic white girls aged 5 to 9 years (47,72,79). Laboratory studies of preschool-aged children have also demonstrated that restrictions placed on children's access to palatable, energy-dense foods can lead to increased food intake when restrictions are lifted and food becomes available (80,81). Other studies, however, found no link between feeding restriction and EAH (54). In many ways, inconsistencies in the literature on EAH parallel those observed in the general literature on child feeding, which may reflect the early stage of the work in the field. Knowledge of child feeding has largely evolved from laboratory studies that address cause and effect but provide limited insight on the usual environments and social interactions surrounding children's behavior. The approach parents take to feeding their children reflects their goals for their children's eating and health, and these goals are influenced by culture and socioeconomic status (82). To some extent, the effect of child feeding practices on children's health requires careful consideration of context.

## Middle Childhood

Another critical period for the development of obesity is during middle childhood. BMI tends to decrease during early childhood and then, typically between the ages of 6 and 8, begins to rise again (adiposity rebound). Excessive rebound and early rebound (before age 5) are related to higher BMI in adulthood (83). An early rebound may reflect the child's taking more control of intake, exposure to gestational diabetes, or early maturation (84).

Children's patterns of weight gain vary by sex and age (85), and during stages of rapid growth, caloric requirements increase. These stages are opportunities for interventions to prevent obesity by controlling caloric intake and increasing energy expenditure. In addition, because the prevalence of obesity increases among children after puberty, as the age of sexual maturity decreases in the population (86) obesity will probably become more prevalent among elementary school students.

Children of school age are highly susceptible to environmental stimuli such as marketing and food availability. Studies suggest that children who are exposed to food advertisements eat more. Several studies of advertising on children's television programs found that the foods promoted increased the risk of becoming obese. In 1 study, at least half of food advertisements during children's television programming were for energy-dense, low-nutrient foods such as cereal, candy, snacks, soda, and fast food (87). Not only do such advertisements promote eating, but eating while watching television also often leads to overeating because children do not notice how much they are eating (88). A recent study showed that for each hour of television watched, children consumed an extra 167 kcal/d (89). This susceptibility is mainly because decision making, critical thinking, and abstract thinking are underdeveloped in childhood. For example, children in Piaget's preoperational stage of cognitive development (ages 2-7) are characterized by illogical and egocentric thought, while children in the concrete operational stage (ages 7-11) cannot think abstractly, reason logically, or make inferences based on available information (90). Exposure to advertisements would decrease by restricting food advertisements that target children, especially during times, such as Saturday mornings, when many children are watching television (87).

The school environment is an opportunity for study and intervention in children's health behaviors because multiple factors can influence obesity in this context. For example, the Coordinated School Health Program model from the Centers for Disease Control and Prevention proposes a multilevel approach in which 8 different school components (eg, health education, nutrition services, healthy school environment, family and community involvement) interact to influence student health (91).

Few studies of school eating patterns have focused on kindergarten and early elementary years, but in third grade, school lunch choices begin to influence children's overall diets (92). Schools' food policies affect student BMI; in 1 study, as the number of school food policies increased, students' mean BMI decreased (93).

The US educational system has typically fallen short in considering health a priority for academic emphasis or outcomes. Health outcomes must be included in the educational agenda and become part of school accountability to obtain support and funding for health-based policies and interventions, such as physical education, comprehensive health education, or BMI monitoring. A survey of the 100 largest school districts in 2006 found that, among the local wellness policies implemented, 99% dealt with nutrition standards of school meals, 97% required nutrition education for at least some grades, and 65% set standards for when teachers can use food to reward children for good behavior or academic accomplishments (94). School programs intended to alleviate the obesity crisis need funding, partnerships, and evaluation. In 2007, the Registered Nurses' Association of Ontario set forth school policies that would prevent childhood obesity; these policies included promoting physical education classes for all students, requiring physical education specialists to be involved in physical education classes, selling healthy foods in cafeterias and vending machines, and promoting walking or biking to and from school (95).

## Adolescence

In addition to developmental risks carried from earlier life, by adolescence the cumulative effects of social disadvantage on obesity become apparent. Analysis of data from the National Longitudinal Survey of Youth Child-Mother File found that having an unmarried mother increased the risk for adolescent overweight. Education and current income were not significantly associated with adolescent overweight, and lifetime income was only marginally significant (21).

The Growing Up Today Study found that subjective social status in the school environment predicted BMI in adolescent girls (96). Girls who ranked themselves at the low end of school social status were 69% more likely to have a BMI 2 kg/m^2^ higher than that of girls of higher subjective social status. The authors concluded that higher subjective social standing in school might protect against weight gain in adolescent girls. The feedback loop in which low self-esteem increases the risk of overweight and overweight contributes to low self-esteem could be a critical point of intervention.

An analysis of data from 12,067 people in the Framingham Heart Study revealed an association between people's weight gain and weight gain in their social networks (97). Rather than occurring randomly throughout social networks, people with BMI ≥30 kg/m^2^ were clustered in the 32-year data set. This finding was not explained solely by social ties between people who were already obese. The chance of becoming obese during a given period increased with development of obesity in a friend (57% increase), a sibling (40% increase), or a spouse (37% increase). The effect was stronger between same-sex friends and siblings. This association did not extend to neighbors, nor was it associated with changes in smoking behavior. A more recent study examined the peer effects on adolescent BMI by using the National Longitudinal Study of Adolescent Health (98). This study found an effect of social networks on obesity in adolescents, and the effect was more pronounced among girls and heavier adolescents. Social networks seem to play a role in the spread of obesity in both adults and adolescents. Therefore, programs that target peer norms may be effective in preventing overweight in adolescents.

Cognitive development during adolescence should also be considered. Although the developmental stage of formal operational thought enables skills such as enhanced problem solving, decision making, and abstract reasoning (90), the underdeveloped prefrontal cortex (99) still leaves adolescents at risk for behaviors that may increase the risk of obesity. For example, adolescents tend to be prone to impulsivity and the fallacy of invincibility and to have problems considering long-term consequences of their behavior. These factors can contribute to poor judgment when it comes to food selection and other health-related behaviors.

## Conclusions

Traditional interventions for child and adolescent obesity often focus on the individual child, with or without family involvement, and include education, modification of diet, and increases in physical activity. Cognitive behavioral strategies are often used to help children make better decisions, solve problems, and monitor their own progress. A recent movement suggests minimizing screen time for youth because television, computers, and video games contribute to sedentary behavior. However, few obesity interventions show clinically or statistically significant weight loss beyond the intervention period (100), which suggests that new and more comprehensive interventions are needed.

To counteract the growing incidence of obesity, interventions must adopt an approach that grasps the interplay of economic, social, behavioral, biomedical, and environmental influences. Such an approach would have to encompass emerging knowledge about how obesity is the consequence of complex factors acting at many stages throughout a person's lifetime. The challenge of intervening in the obesity epidemic becomes even more daunting with the realization that, in children and adolescents, these influences must be considered separately at each stage of development.

This overview of the developmental influences on childhood overweight and obesity suggests opportunities for intervention. To combat prenatal influences on child obesity, pregnant women should be strongly discouraged from smoking and encouraged to consume a diet low in fat with adequate protein. Additionally, breast-feeding may decrease the risk of overweight, particularly in children born to overweight mothers. Because women are more motivated to change behaviors during pregnancy and immediately postpartum, these intervals can be targeted to shape eating patterns of both mother and child.

Interventions to prevent child and adolescent obesity should focus on multiple settings, including the home and school. Further research should investigate variables in family relationships, the home, and the extended environment that influence eating. Schools can intervene by offering healthy food choices in their breakfast and lunch programs and vending machines. Empowering families and schools and giving them resources to engage in obesity prevention efforts and to provide environments that support healthy behaviors are critical issues that governments and social institutions need to address.

## Figures and Tables

**Table.** Risk Factors for Obesity in Childhood and Adolescence

Variable	Gestation	Early Childhood (Birth Through Age 5 y)	Middle Childhood (Ages 6-12 y)	Adolescence (Ages 13-18 y)
**Biological development**	• Risk for metabolic syndrome increases with exposure to glucocorticoids, protein restriction, maternal diet and obesity • Exposure to food flavor in utero • Maternal smoking	• Heightened preferences for sweet, salts, fats; rejection of bitter • Heightened sense of smell • Variation in taste receptor genes • Breast-feeding, exposure to food flavor in breast milk • Low birth weight and BMI rebound • Weaning process • Portion size and meal timing • Conditioned food preferences, associative learning	• Adiposity rebound • Conditioned food preferences • Portion size	• Change in composition of body mass (fat and nonfat tissue) • Change in distribution of fat • Portion size
**Cognitive development**	NA	NA	• Concrete operational thought • Decision making	• Formal operational thought (abstract thought) • Decision making and problem solving • More prone to impulsivity • Invincibility • Problems considering long-term consequences of actions
**Psychosocial development**	NA	• Parental feeding practices, family mealtime routine • Presence of adult, modeling during feeding • Foods develop sociocultural meaning • Exposure to media • "Balance of power": children strive for increasing autonomy and control	• Parental feeding practices, family mealtime routine • Presence of adult, modeling during feeding • Peer influences • Foods develop sociocultural meaning • Increasing exposure to media • Development of body image • Eating outside the home • Quality of school food/vending	• Family mealtime routine • Peer influences, greater social network influences • Foods develop sociocultural meaning • Increasing exposure to media • Heightened awareness of body image • Eating outside the home • Quality of school food/vending • Coping with stress • Style of intake control (eg, dieting, eating disorders, disinhibited eating)
**Intervention strategies that have been tried**	• Promote breast-feeding • Encourage healthy prenatal nutrition	• Increase parenting skills and teacher involvement in teaching healthful behaviors • Increase fruit, vegetable, fiber consumption • Encourage meals at home • Increase daily activity/exercise	• Involve family in treatment • Use age-appropriate dietary modification • Reduce screen time • Use behavior-based strategies and curricula • Increase opportunities for physical activity and availability of healthy foods at schools	• Involve family in treatment • Reduce caloric intake and increase physical activity • Reduce screen time • Use behavior-based strategies and curricula • Increase opportunities for physical activity and availability of healthy foods at schools

Abbreviations: BMI, body mass index; NA, not applicable.
